# Evaluating the Impact of Dropout and Genotyping Error on SNP-Based Kinship Analysis With Forensic Samples

**DOI:** 10.3389/fgene.2022.882268

**Published:** 2022-06-30

**Authors:** Stephen D. Turner, V.P. Nagraj, Matthew Scholz, Shakeel Jessa, Carlos Acevedo, Jianye Ge, August E. Woerner, Bruce Budowle

**Affiliations:** ^1^ Signature Science, LLC., Austin, TX, United States; ^2^ Center for Human Identification, University of North Texas Health Science Center, Fort Worth, TX, United States; ^3^ Department of Microbiology, Immunology, and Genetics, University of North Texas Health Science Center, Fort Worth, TX, United States

**Keywords:** SNP, kinship, forensics, genealogy, forensic genetic genealogy, relatedness

## Abstract

Technological advances in sequencing and single nucleotide polymorphism (SNP) genotyping microarray technology have facilitated advances in forensic analysis beyond short tandem repeat (STR) profiling, enabling the identification of unknown DNA samples and distant relationships. Forensic genetic genealogy (FGG) has facilitated the identification of distant relatives of both unidentified remains and unknown donors of crime scene DNA, invigorating the use of biological samples to resolve open cases. Forensic samples are often degraded or contain only trace amounts of DNA. In this study, the accuracy of genome-wide relatedness methods and identity by descent (IBD) segment approaches was evaluated in the presence of challenges commonly encountered with forensic data: missing data and genotyping error. Pedigree whole-genome simulations were used to estimate the genotypes of thousands of individuals with known relationships using multiple populations with different biogeographic ancestral origins. Simulations were also performed with varying error rates and types. Using these data, the performance of different methods for quantifying relatedness was benchmarked across these scenarios. When the genotyping error was low (<1%), IBD segment methods outperformed genome-wide relatedness methods for close relationships and are more accurate at distant relationship inference. However, with an increasing genotyping error (1–5%), methods that do not rely on IBD segment detection are more robust and outperform IBD segment methods. The reduced call rate had little impact on either class of methods. These results have implications for the use of dense SNP data in forensic genomics for distant kinship analysis and FGG, especially when the sample quality is low.

## Introduction

Inferring familial relationships between individuals using genetic data is a common practice in population genetics, medical genetics, and forensics. Advances in next-generation sequencing (NGS) and genotyping microarray technology have enabled the rapid profiling of millions of single nucleotide polymorphisms (SNPs) with near-perfect accuracy. With these new methods, investigators have improved one of the most significant challenges in forensic analysis: attribution and identification of the source or close relatives of DNA samples from unknown donors. In response to these advances, SNP-based kinship analysis algorithms and software for analyzing and inferring genetic relationships between individuals have been developed.

The methods for SNP-based kinship analysis and relatedness inference on genome-wide data can be broadly classified into two categories: 1) genome-wide relatedness methods and 2) identity by descent (IBD) segment–based relatedness estimation methods. Likelihood methods are also useful when working with data more sparse than microarray or whole-genome sequencing data (Kling and Tillmar, 2019). Generally, genome-wide relatedness methods have lower data and resource requirements, requiring only a sparse set (e.g., <20,000) of SNPs in linkage equilibrium to perform as well as with genome-wide data and allow the use of unphased genotypes and low density of variant calls. By contrast, IBD segment methods usually (but not always, e.g., IBIS (Seidman et al., 2020)) require dense phased data and would require the imputation of incomplete genotyping or SNP data to fill in missing genotypes (or removal of sites with missing data in any sample). With respect to applications and assuming high-quality (low-error) genotyping data, genome-wide relatedness methods perform as well as IBD segment methods for close relationships, while IBD segment methods more accurately identify more distant relationships (Ramstetter et al., 2017).

The goal of this study was to evaluate the accuracy of genome-wide relatedness methods and IBD segment approaches in the presence of challenges that are commonly encountered with forensic data, namely, the high level of dropout (low call rate) and increased genotyping error. This study differs from Gorden et al. (2022), where we simulate genome-wide microarray rather than SNP capture; this differs from similar work in de Vries et al. (2022) by using different simulation strategies, different pedigree and error structures, and uses both crossover interference and a sex-specific genetic map for forward-time simulation. The challenges with forensic samples are commonly encountered due to samples being degraded, contaminated, and/or having limited input from trace amounts of DNA. Furthermore, in disaster victim identification scenarios, DNA from *all* samples, queries, and references may be of low quality. To this end, a selection of tools was identified based on previously published data that fit into either of these categories of relatedness analysis methods to assess for relatedness characterization. Systematic and comprehensive benchmarking requires many pedigrees with known relationships, which can be generated across a range of call rates and genotyping error, using multiple populations with different biogeographic ancestries. As such, developing a comprehensive set of benchmarking data using simulation was an equally important aim of this study. The performance of different kinship identification methods and software implementations was assessed for accurately quantifying relatedness using the simulated data generated within this study. The specific goal for this study was to benchmark the performance of different approaches to SNP-based kinship analysis to detect and characterize first through third-degree relatives in the presence of challenges associated with forensic genetic data.

## Methods

### Genome-Wide Relatedness and Identity by Descent Segment Detection

There are several existing methods for the inference of relatedness from SNP data (Ramstetter et al., 2017). The methods for relatedness inference were classified into two broad categories: genome-wide relatedness measures and IBD segment detection methods. An additional goal of the systematic review conducted here was to identify datasets and/or simulation methods that can be used to benchmark the performance of algorithms and software for SNP-based kinship analysis. Many of the publications describing the selected methods performed their own benchmarking with empirical or simulated data; these published benchmarking datasets were assessed for their utility in the testing and evaluation goals of this study. Testing and evaluation require related individuals with known ground truth pedigree information, and these kinds of data can be created *in silico* by sampling existing well-characterized unrelated individuals such as those available in the 1000 Genomes Project (Auton et al., 2015) under a forward-time simulation framework. As such, this systematic evaluation included existing methods and software implementations for simulating genome-wide SNP genotyping data.

The KING algorithm (Manichaikul et al., 2010) was chosen as an exemplar genome-wide relatedness method as it allows for the presence of unknown population substructure, enabling the robust estimation of the kinship coefficient, regardless of sample composition, and does not require the specification of allele frequencies for calculating the kinship coefficient, which may not be known prior to analysis. Two different IBD segment approaches were selected. IBIS (Saada et al., 2020) allows for the IBD segment inference from dense genotyping data without the requirement for phased data. In addition to reduced resource requirements, a phase-free inference may be robust to phasing errors that can be introduced with genotyping data and small sample sizes. In addition, hap-IBD (Zhou et al., 2020), which requires phased inputs, has recently been demonstrated to enable fast, scalable, and accurate IBD segment detection in comparison to other leading methods.

When attempting to rescue the performance of IBD segment approaches, more permissive parameter settings were used with both IBIS and hap-IBD (described in Supplementary Table S2). A thorough description of methods and selection criteria can be found in the Supplementary Data.

### Reference Genotypes

Founder haplotypes were obtained from the 1000 Genomes Project (GRCh37). The set of variants was thinned to 590,588 autosomal biallelic SNPs primarily those represented on the Illumina Global Screening Array (GSA) (see Supplementary Data), a commonly used platform for medical genomics and forensic genetic genealogy (FGG) analysis. In addition to capturing a large set of known polymorphic sites (including sites that confer high imputation accuracy in non-European populations), this array-centric strategy is commonly used in the genetic genealogy space even when whole-genome sequencing is used to generate data. Even though the GSA is by no means historically the only array used to generate data that populates forensic genetic genealogy (FGG) databases, it is a commonly used platform for medical genomics and FGG analysis.

### Data Simulation

Pedigrees and phased genotypes for benchmarking of the selected tools were simulated using ped-sim (Caballero et al., 2019), using the keep_phase flag, to allow direct comparison between tools. Ped-sim outputs IBD segment sizes and positions from an input pedigree definition. When supplied with population genotype data from a pool of founders, ped-sim will use forward-time genetic data simulation to generate genotype data in simulated pedigrees, allowing specified error rates to be introduced into the output data. The effect of removing SNPs from original 1000 Genomes genotypes was investigated by performing a limited number of simulations using identical family structures with both the full genotypes and the selected SNP genotypes and performed an accuracy assessment. The effects of SNP selection on the kinship calculation did not materially affect the overall relationship between the tools and their accuracy, indicating that using a reduced SNP set was a reasonable proxy for the wider pools of SNPs (Supplementary Data).

A total of 264 simulations were created using unrelated founders from each of three populations in 1,000 Genomes: GBR (British in England and Scotland), ASW (African Ancestry in Southwest US), and MXL (Mexican Ancestry in Los Angeles, California), respectively. Each of the three population’s data was simulated using a range of missing genotype and genotyping error rates. The full details of simulation are provided in the Supplementary Data. Sweeping through each of the parameter combinations earlier resulted in 88 simulations per population for a total of 264 simulations across the three populations studied across a wide range of missingness and genotyping error rates (see Supplementary Table S1). For each simulation, five-generation pedigrees were simulated (Supplementary Figure S1), resulting in a wide range of relative types: 130 first-degree relationships, 184 second-degree relationships, 212 third-degree relationships, 212 fourth-degree relationships, 176 fifth-degree relationships, 144 sixth-degree relationships, 64 seventh-degree relationships, and 769 unrelated pairs for each pedigree. The counts and distribution of these relationship degrees are shown in Supplementary Figure S2.

### Benchmarking

The accuracy of each approach was measured by comparing the calculated degree of relatedness to the true relatedness degree from the simulated pedigree. In all categorical analyses, relationships were classified as first through the fourth degree, with anything more distant than the fourth degree binned as “unrelated,” and accuracy was assessed using classification accuracy. The relationship degree for IBIS used the built-in degree inference. KING and hap-IBD relationship degree inference used conventional cutoffs (geometric means between theoretical medians for each degree) as described in Manichaikul et al. (2010). Quantitative accuracy analysis was also calculated by comparing the *estimated* kinship coefficient to the *actual* kinship coefficient that was simulated using the root mean square error (RMSE).

## Results

### Performance on Simulated Pedigrees With Missing Data and Genotyping Error

**FIGURE 1:**
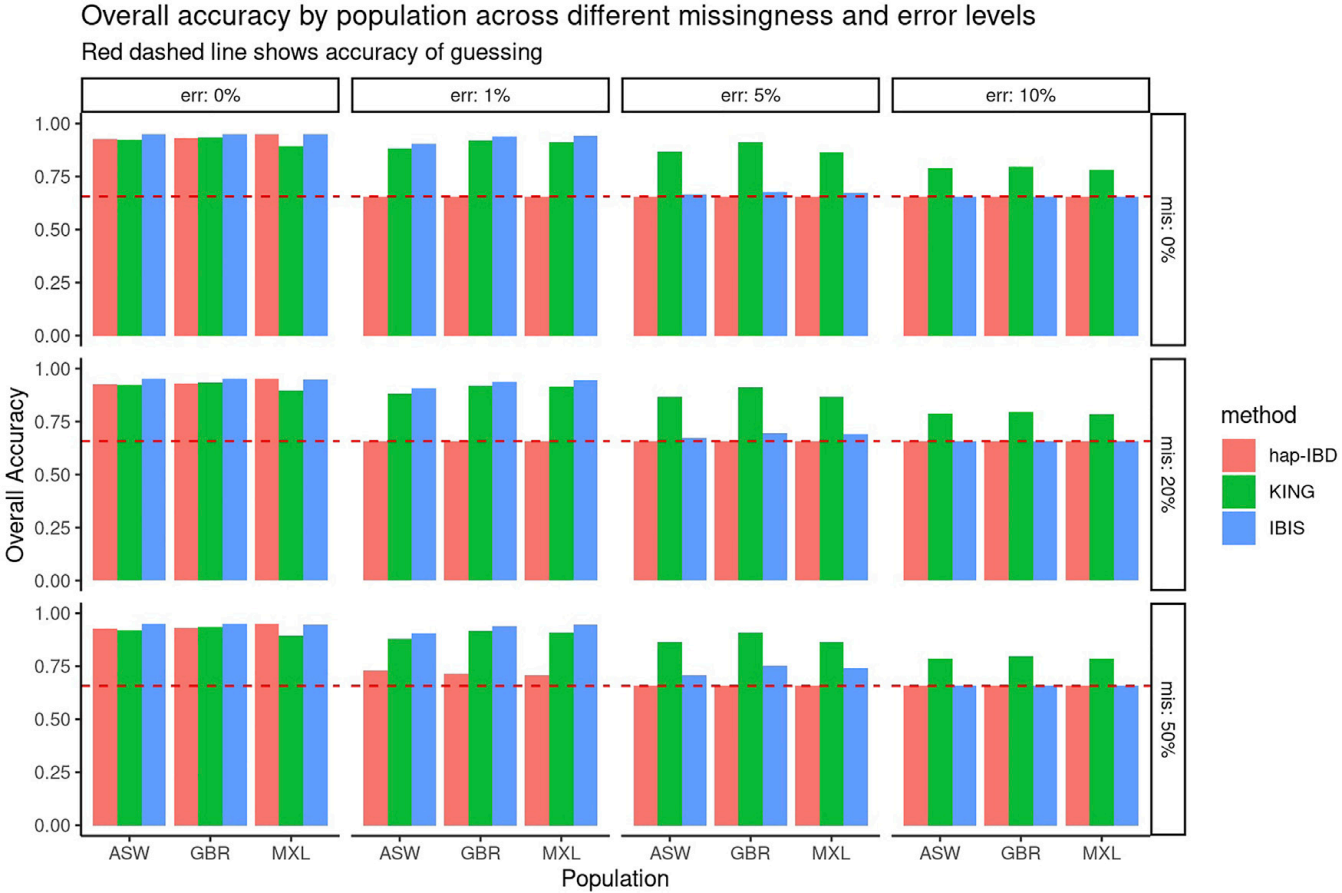
Overall classification accuracy using default parameters. Panels show the genotyping error increasing in panels going left-to-right and missing data rates increasing panels going top-to-bottom. Individual bars within each panel show the classification accuracy within each simulated population. This graphic shows roughly equivalent accuracy with zero error but decreased accuracy for both IBD segment methods in comparison to KING with a higher genotyping error.

**FIGURE 2:**
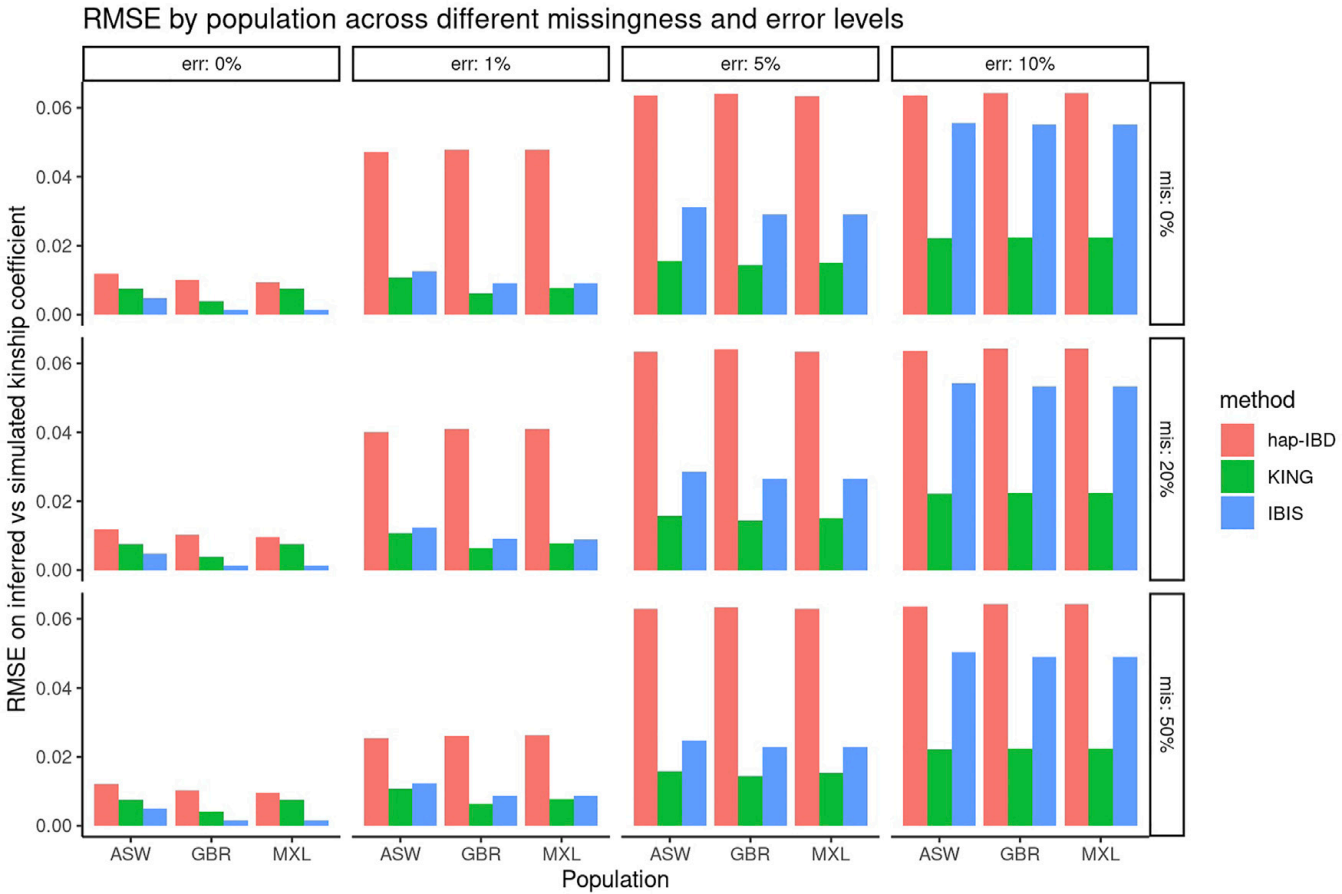
RMSE comparing the inferred versus simulated kinship. Panels show the genotyping error increasing in panels going left-to-right and missing data rates increasing in panels going top-to-bottom. Individual bars within each panel show the classification accuracy within each simulated population (ASW, GBR, and MXL).

First, the performance of KING, IBIS, and hap-IBD was assessed against data with increasing missingness and the genotyping error, using their default settings without any parameter tuning. To align with analysis most useful for forensic genealogy investigations (Greytak et al., 2019; Gorden et al., 2022), classification accuracy was assessed focusing on identifying relatively close relatives; therefore, relationship degree classification resolution was limited to the fourth degree, where any relationship more distant than the fourth degree was classified as “unrelated.” This aligns most closely with the use case of missing persons’ identifications, where identifications are made with relatives closer than the fourth degree. Figure 1 shows classification accuracy for KING, IBIS, and hap-IBD against the simulations generated using ASW, GBR, and MXL genotypes from 1000 Genomes. Classification accuracy is near 100% for all populations using all kinship methods when there is zero genotyping error. Missing data, without the genotyping error, has little to no effect on overall classification accuracy. Classification accuracy decreases for all methods as the genotyping error increases. Notably, the classification accuracy for both IBD segment methods, IBIS and hap-IBD, falls near the accuracy of guessing, as the error increases (indicated by the dashed red line, See Supplementary Data). IBIS accuracy decreases to near the accuracy of guessing when the error is between 5 and 10%. The accuracy of hap-IBD approaches this point when the genotyping error is ≥1%. By contrast, the classification accuracy for KING is still impacted by the genotyping error but not to the degree that the IBD segment methods are affected. We also assessed accuracy quantitatively by evaluating the RMSE of the *estimated* kinship coefficient compared to the *actual* kinship coefficient for each pair of relatives as simulated, shown in Figure 2. These results exhibit a similar trend as the classification error results described earlier; increasing missing data rates do not noticeably increase any method’s RMSE. Increasing the genotyping error, however, has a substantial effect. The RMSE for KING is slightly higher than that of IBIS or hap-IBD when the genotyping error is zero, but it is impacted less dramatically at higher levels of the genotyping error. By contrast, both IBD segment methods have much higher RMSE at higher levels of the error in comparison to the KING genome-wide relatedness method. Increasing error rates give rise to higher RMSE as the genotyping error increases. Next, the correlation between the calculated and simulated kinship coefficient was examined using a single population (GBR) under default parameters for each method. Supplementary Figures S3–S5 show the estimated kinship coefficient compared to the simulated kinship coefficient for KING, IBIS, and hap-IBD, for each pair of relatives simulated. These results indicate that with zero genotyping error, there is no systematic bias, but IBD segment methods show substantially less variability. As the genotyping error increases, the kinship coefficient inferred by all methods is underestimated compared to the true kinship coefficient between two individuals. The underestimation of the true kinship coefficient is much more dramatic in the IBD segment methods when using default parameters. In summary, these results demonstrate that while KING has higher variance than IBD segment methods in the absence of genotyping error, the KING method is more robust than either of the two IBD segment methods as the genotyping error increases. These results also indicate that missing data have little to no effect on the ability of any of these methods to assess relatedness, given dense genotype data.

**FIGURE 3:**
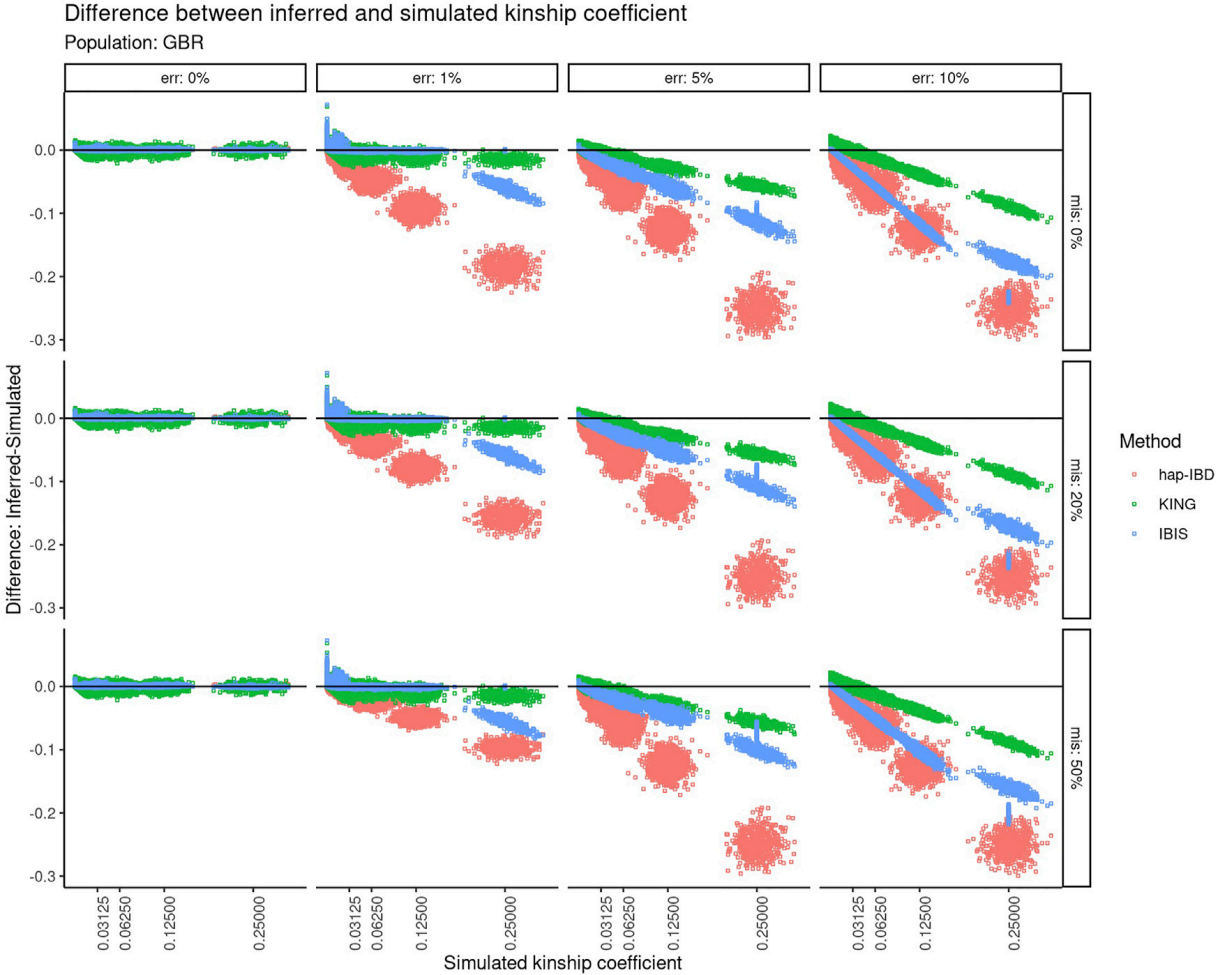
Difference between the inferred kinship coefficient versus the true simulated kinship coefficient for three different methods using default parameters at different error and missingness levels for simulated relationships from GBR founders. Error increases in panels going left-to-right. Missing data increase in panels going top-to-bottom. Each point represents a pair of simulated individuals. Red = hap-IBD; green = KING; blue = IBIS.

The calculated kinship coefficient was compared to the recorded simulated kinship coefficient for all pairwise comparisons. Figure 3 shows the difference between the calculated and simulated kinship coefficient as a function of the increasing simulated kinship coefficient. A perfect correspondence between the estimated kinship coefficient versus the kinship coefficient that was simulated would be displayed as a horizontal line centered at zero on the *Y*-axis. With zero genotyping error, there is very little difference between the estimated versus simulated kinship coefficient, regardless of the recorded kinship coefficient of the two individuals compared. As the genotyping error increases, the negative impact on relationship estimation becomes clear for KING and both IBD segment methods. All three methods suffer from an increasingly underestimated kinship coefficient as the true relationship is closer, but the degree to which these methods are impacted differs highly. At 1% error, KING and IBIS are less impacted at closer relationships, but IBIS is impacted more negatively for closer relationships. At higher levels of error (5–10%), both IBD segment methods are much more severely impacted than KING, especially with closer relationships. The performance of hap-IBD was extremely poor with any genotyping error ≥1%.

### Assessing the Impact of Identity by Descent Segment Method Parameters With High Genotyping Error

The aforementioned results indicate that when used with default parameters both IBD segment methods are extremely sensitive to the genotyping error compared to the KING estimator. These methods were developed and optimized primarily for use in population or medical genetics studies where there is a reasonable expectation for accurate and robust data. With high-quality samples, industry-standard microarray genotyping or short-read sequencing and variant calling routinely produce data with well over 99.9% accuracy (Olson et al., 2021). These methods were developed using sensible defaults that maximize the ability to detect long IBD stretches minimizing artificial breaks introduced by sequencing or genotyping errors, all while minimizing false positives that may be introduced while being too permissive. The KING method does not have parameters that can be tuned. However, IBIS and hap-IBD can be run with different parameters. For tools that calculate IBD segments, the most impactful measures are related to the minimum length of segment reported and the maximum distance between SNPs for a segment to be continued. With IBIS, the allowed error rate, which specifies the acceptable number of mismatches in a segment before the extension of a segment is stopped, the minimum number of shared SNPs required to identify a segment, and the minimum length of any detected segments to output were all varied. The error rate tolerance was increased up to 0.2 (20%), the minimum SNPs required to identify a segment were decreased to a minimum of 2, and the minimum length decreased to a minimum of 2 cM. With hap-IBD, the max gap parameter, which specifies the maximum base-pair gap between a seed segment and another IBS segment in order for the seed segment to be extended (default 1,000), was varied. The gap value was increased to 5,000 and 10,000, which allows output IBD segments to include very short non-IBS regions that can result from the genotype error, mutation, and gene conversion. Also varied were hap-IBD’s minimum output length from the default of 2 cM to add more permissive (1 cM) and more restrictive (7 cM) options.

In the results presented earlier, the tool parameters were held constant at defaults, and the performance was examined over different missing and genotyping error rates. Here, the simulation constant was held at zero missing data. For IBIS, the simulated data generated by ped-sim using 5% simulated error were used. For hap-IBD, due to its higher sensitivity to the error, ped-sim simulations allowing the 1% error were selected. These error rates are the last simulation for each tool with accuracy significantly greater than the accuracy of guessing.

Permissive parameters allowing the variation of acceptable IBD segments were not able to rescue the performance of IBD segment methods assessed here. As mentioned earlier, the correlation between the estimated and simulated kinship coefficient was examined when using more permissive IBIS and hap-IBD parameters. These results indicate that the variation of IBIS parameters (allowed mismatches in a segment before extension, and the minimum length of detected segments) does not improve kinship coefficients for high error data and that varying the minimum segment length for hap-IBD results in severely underestimated kinship coefficients, regardless of the allowed error.

In summary, increasing the acceptable error rate in a segment before considering it as false and decreasing the minimum centimorgan length to consider a segment IBD did not have an appreciable impact on increasing the kinship inference accuracy in the presence of high genotyping error for either IBIS or hap-IBD. Taken together, these results indicate that no combination of reasonably permissive parameters could rescue the performance of the two IBD segment methods assessed here when the genotyping error is in the 1–5% range.

## Discussion

Dense SNP genotype data have made it possible to infer relationships between samples with far greater granularity than what is possible using traditional short tandem repeat (STR) analysis with capillary electrophoresis. The methods for measuring genome-wide relatedness (Purcell et al., 2007; Manichaikul et al., 2010; Conomos et al., 2016) and for detecting IBD segments (Gusev et al., 2009; Browning and Browning, 2011, 2013; Caballero et al., 2019; Naseri et al., 2019; Saada et al., 2020; Zhou et al., 2020) are routinely used for characterizing relatedness in medical genetics, genome-wide association studies, population genetics, and, more recently, forensics.

Recent benchmarking efforts have shown that genome-wide relatedness methods and IBD segment methods performed equally well (assuming little or no error) and are generally accurate for close relationships, but IBD segment methods outperformed genome-wide relatedness methods for more distant relationships (Ramstetter et al., 2017). The goal of the present study was to assess how these methods perform in the special case of forensics, which differs from other common use cases in population genetics and public health genomics. In forensics, and especially in the field of forensic genetic genealogy (FGG) (Greytak et al., 2019), the samples are rarely the same degree of quality and quantity as is usually available in population and medical genetics. Instead, the samples are often degraded, and in some cases, DNA is present in only minute, trace amounts. The combination of low input together with degraded template DNA can lead to both increased missingness (low call rate for microarrays or high dropout rate for sequencing) and an increased genotyping error (Alaeddini et al., 2010; Loreille et al., 2011; de Vries et al., 2022). All the methods discussed in this work were developed and optimized for contemporary genotyping array data or variant calls from short-read whole-genome sequence data, both of which have extremely high accuracy and low missing data rates, given high coverage and high-quality samples. This study aimed to assess how genome-wide relatedness methods and IBD segment methods perform with dense genotyping data in the presence of integrity challenges typically seen with forensic samples.

The initial analysis focused on assessing the performance of a commonly used genome-wide relatedness measure (KING) and two IBD segment methods (IBIS and hap-IBD). Of the many IBD segment methods reviewed in this study, the focus was on two methods to thoroughly benchmark: IBIS and hap-IBD. IBIS does not require phasing, and in the forensics use case having very few samples available, phasing could introduce an additional error that would degrade the performance of IBD segment detection. The software hap-IBD was chosen based on its independent benchmarking against other highly cited and well-known IBD segment methods because of its permissive licensing.

The analysis of classification accuracy and RMSE indicated that missing data had scarcely any noticeable effect on the performance of the tools evaluated. With zero genotyping error, both IBD segment methods outperformed KING. With no error, all methods showed no systematic bias while both IBD segment methods showed substantially less variance than KING. However, this increased performance was small, in that the accuracy of KING was still relatively high—the absolute difference in RMSE between KING and either IBD segment method was small. In the typical use case of, for example, missing persons’ identification where a sample is attempted to be putatively placed into a pedigree containing close relatives (i.e., first-, second-, and third-degree relatives), this increased performance may be inconsequential. The ability of IBD segment methods to accurately discriminate between more distant relatives (e.g., fifth degree and beyond) where genome-wide methods such as KING are only reasonably accurate to a third or fourth degree has been demonstrated previously (Ramstetter et al., 2017), and our results are consistent with those previous observations. Although identification of very distant relatives may be an aim of FGG (Greytak et al., 2019), which will be discussed further in the following section, the use case here is the accurate identification of close relationships. To this end, all methods performed similarly well when no genotyping error was present. This observation suggests that when using high-quality reference samples on all individuals, the relatively low-resource requirements of a method such as KING may be preferable to other methods which may require phasing, imputation, or other preprocessing. However, if identification of more distant relationships is of interest, IBD segment methods have a notable advantage over any genome-wide method.

The introduction of genotyping error changes these aforementioned conclusions dramatically. All of the results shown here clearly demonstrate that the genotyping error causes a serious and apparently irrecoverable drop in accuracy for both IBD segment methods far more than the drop in accuracy seen with KING. The accuracy of hap-IBD falls to the lowest possible classification accuracy and assigns nearly all samples as unrelated when any error (≥1%) is introduced. At 5% error and above, the performance of either of the IBD segment methods is demonstrably diminished. The genotyping error also affects KING and notably so at high error rates (5% and especially 10%), but the reduction in accuracy is far less than what is seen in either of the IBD segment methods. As such, this first set of experiments clearly shows that the IBD segment methods assessed here are extremely sensitive to any genotyping error when used with default parameters. Furthermore, we simulated data keeping true phase, which means that phasing errors were not introduced by a phasing step on unphased data (which will be required for practical FGG applications), mimicking the best-case scenario for phasing. The reasonable ranges of more permissive parameter settings were assessed in either of the IBD segment methods used here to determine if the performance of these methods could be rescued in the presence of the genotyping error profiles expected with degraded forensic samples. Even the most permissive (yet still reasonable) parameter combinations could not rescue the performance of either of these methods. Any further increase in the permissiveness of these parameters would almost certainly result in false-positive IBD segments being reported for cases where the error was not as high as simulated here.

These observations have several notable implications for the use of dense SNP genotype data in a forensics context. First and foremost, the genotyping error that might be common in low-input or degraded samples will almost certainly result in missed relationship identification. This consequence is particularly true if an IBD segment method is used when the genotyping error is >1%. The genome-wide KING method is more robust to the genotyping error but still suffers from the underestimation of the kinship coefficient between two samples when the error rate is high, and the degree of this underestimation increases with closer relationships. Perhaps more broadly notable, the findings here have serious implications for the current state-of-the-art in FGG. In FGG, DNA is extracted from the remains of an unidentified person, or DNA is collected from an evidence sample, from an unknown donor, which was left behind at a crime scene. This DNA sample is subjected to dense microarray SNP genotyping or whole-genome sequencing and is typically uploaded into a third-party database such as GEDmatch, where distant relatives are identified using IBD segment methods (Greytak et al., 2019). The data here show that IBD segment analysis approaches become unreliable between 1 and 5% error, creating a risk that FGG will fail to identify true relatives, and may falsely identify unrelated individuals as candidate matches. Our simulations contained the specified level of error in *all* samples, not just the query sample; if all reference samples were of high quality but only the query forensic sample was of lower quality, the performance degradation may be mitigated to some degree. However, here, we took a more challenging approach—with poor-quality samples being compared to other poor quality samples common in disaster victim identification or other mass casualty events (Prinz et al., 2007; Watherston et al., 2018; Bertoglio et al., 2020) to address a “worst-case scenario.”

Future research in this area should prioritize further exploration and more extensive evaluation of IBD segment methods with a specific focus on those that have parameters that allow more explicit fine-tuning of parameters relating to IBD segment seeding and extension, with the goal of identifying a method or algorithm for rescuing the performance of IBD segment detection in the presence of high error. Furthermore, the scope of the present study only examined the performance of relative matching techniques using called *SNP genotypes*, which could be from either microarray SNP genotyping or from whole-genome sequencing that have already gone through alignment and variant calling to generate a VCF with called SNP genotypes. Investigating methods that operate directly on *genotype likelihoods* was out of the scope of the current study. Recently published methods including NgsRelate (Korneliussen and Moltke, 2015) and NGSremix (Nøhr et al., 2021) operate directly on genotype likelihoods. Future research should assess how these methods perform when dealing with low-quality sequencing data from degraded or low-quantity sequencing data.

## Data Availability

The datasets presented in this study can be found in online repositories. The names of the repository/repositories and accession number(s) can be found at: https://zenodo.org/record/6079161.
