# Supplementary material for: Evaluating the Impact of Dropout and Genotyping Error on SNP-Based Kinship Analysis With Forensic Samples
Source: Front Genet. 2022 Jun 30;13:882268. doi: 10.3389/fgene.2022.882268 (PMC9282869; doi:10.3389/fgene.2022.882268)
Supplement: Supplementary file 1 [file DataSheet1.pdf]

frontiers

Supplementary Material

1 Supplementary Data

1.1 Supplementary methods 1: method review and selection criteria.

The scope of the methods review described in the main text includes existing methods for simulating dense genotype data and existing methods for SNP-based kinship analysis of dense genotype data (i.e., variant calls in VCF format (Danecek et al., 2011)). While additional components may be necessary to generate data needed to feed these methods (e.g., alignment and variant calling of WGS data, genotype phasing, genotype imputation, methods for low-coverage WGS, etc.), these methods are out of scope of this review. Finally, while this review does cover implementations that are restrictively licensed, only permissively licensed (e.g., GPL, MIT, BSD, Apache) tools and methods were carried forward to later tasks. Noncommercial or academic use only implementations were not further evaluated, nor were their methods reimplemented in this study.

There are two methods examined in detail: genome-wide relatedness, and IBD segment methods. Genome-wide relatedness methods, most frequently implemented using the KING-robust method, estimated genome wide relatedness of samples (Manichaikul et al., 2010). These methods rely on calculations of relatedness and IBD segment sharing across entire genomes simultaneously. Methods of this type are recommended to use population specific allele frequencies, but it is not required to do so. As a result of this methodology, the KING-robust methodology is suited for a wide range of SNP densities. Genome wide relatedness measures have been shown to be effective at prediction of close relatives (3rd degree or closer), with decreasing accuracy as relationships become more distant. More recent IBD segment methods are more sensitive, and able to accurately distinguish between relatives as distant as 6th, 7th or 8th degree. They are also more computationally expensive, often (but not always) requiring phasing and possibly imputation. These methods identify segments of homologous chromosomal SNP data from a seed-extend approach, marking all potential IBD segments with identity to another individual, and calculating kinship coefficient as a function of the genetic distance (in centiMorgans) shared between individuals. To accomplish this, most IBD segment methods rely on accurate phasing of genomic SNP data. This method is more sensitive but requires high-quality data to accurately identify and extend segments.

1.1.1 Genome-wide relatedness: KING

The KING algorithm (Manichaikul et al., 2010) allows for the presence of unknown population substructure, enabling robust estimation of the kinship coefficient in the presence of population structure. Simulation and analysis with real data as benchmarked showed accurate relationship estimation of third degree relatives (Ramstetter et al., 2017). The KING publication showed that the assumption of population homogeneity in PLINK (Purcell et al., 2007; Chang et al., 2015) resulted in biased results, systematically inflating the apparent relatedness degree (kinship coefficient). The publication presented two estimators of the kinship coefficient - one that assumes homogeneity, and another that does not (KING-robust). The authors also found that results of relationship inference on data with population structure were similar using a full set of SNPs as compared to a subset of rare SNPs. The KING software implementation is not permissively licensed and thus was not evaluated any further. Additionally, the KING software only takes PLINK-formatted .bed/.bim/.fam files as input, rather than the more widely used industry standard VCF, and thus does not take advantage of HTSlib for quickly reading and parsing genetic variation data. AKT is an efficient toolkit that re-

implements the KING-robust algorithm in C++ using HTSlib for reading industry standard VCF/BCF (Arthur et al., 2017). AKT takes standard VCF as input, provides a consistent API, and provides a tidy output structure for pairwise kinship coefficients and inferred IBD proportions. The KING-robust algorithm as implemented in AKT has very low resource requirements: its input is a set of SNPs (optionally LD-pruned) and does not require any preprocessing such as phasing or imputation.

1.1.2 IBD segments: IBIS

IBIS (Saada et al., 2020) allows for IBD segment inference from dense genotyping data *without the requirement for phased data.* The publication benchmarked against RefinedIBD (Browning and Browning, 2013), GERMLINE (Gusev et al., 2009), and KING (Manichaikul et al., 2010). Benchmarking included assessing IBD segment identification accuracy, but more importantly, classification accuracy of inferred relationship degrees using both simulated and real data. IBIS accepts PLINK bed/bim/fam as inputs, and outputs a list of IBD segments. Notably, the *phase-free* inference makes IBIS much faster than competing methods and robust to phasing errors that can be introduced with genotyping data and small sample sizes.

1.1.3 IBD segments: hap-IBD

The software hap-IBD (Zhou et al., 2020) enables extremely fast and scalable IBD segment detection. It uses a positional Burrows-Wheeler transform and parallelization to achieve speed and a novel compression method to reduce memory. The publication describes benchmarking against TRUFFLE (Dimitromanolakis et al., 2019), iLASH (Shemirani et al., 2019), and RaPID (Naseri et al., 2019). The benchmarks indicated that hap-IBD has lower false positives than RaPID and higher true positives compared to iLASH, while TRUFFLE has high error rates for short IBD segments. Computationally, hap-IBD was faster than all other methods at large sample sizes, but for smaller samples sizes (e.g. 5,000) there was no appreciable absolute difference.

1.1.4 Other methods not evaluated

Other IBD segment methods that were not evaluated included TRUFFLE (Dimitromanolakis et al., 2019), iLASH (Shemirani et al., 2019), and RaPID (Naseri et al., 2019). These tools are not permissively licensed and as such were not further evaluated. Additionally, other tools such as ERSA (Huff et al., 2011), PRIMUS (Staples et al., 2014), and PADRE (Staples et al., 2016) were not evaluated also because of restrictive licensing. Methods that rely on genotype likelihoods (Korneliussen et al., 2014; Korneliussen and Moltke, 2015; Waples et al., 2019) fundamentally did not rely on called genotypes and were therefore beyond the scope of this evaluation. We furthermore did not evaluate GERMLINE, as other tools assessed here benchmarked with higher accuracy and speed than GERMLINE in other benchmarking studies (Gusev et al., 2009; Browning and Browning, 2011; Zhou et al., 2020).

1.2 Supplementary methods 2: subsetting variants to common array (GSA) sites

We aimed to keep only SNPs represented on a commonly used microarray platform. The Illumina Global Screening Array (GSA) is the most widely used genotyping platform in the direct-to-consumer genomics and ancestry testing space, used by 23andMe, AncestryDNA, Family Tree DNA, and MyHeritage DNA (International Society of Genetic Genealogy, 2021). Accordingly, the overwhelming majority of data in the most comprehensively used genealogy platform, Gedmatch, is derived from the Illumina GSA, and this overrepresentation will become even more pronounced over the coming years. While this effort does not rely on Gedmatch and is not evaluating Gedmatch in any way, if we are to reduce representation of founders' SNP genotype data to a manageable subset that

This is a provisional file, not the final typeset article

mimics data generated on a dense SNP genotyping platform, using the GSA as this backbone is the most sensible place to start. There are 583,089 biallelic SNPs genotyped on the GSA that have genotype data in the 1000 Genomes Project data.

In addition to the sites from the GSA, small panels of kinship-informative SNPs were added. These SNPs include 1,262 “reliable” SNPs for kinship analysis (Arthur et al., 2017) – these SNPs are biallelic SNPs in 1000 Genomes Phase 3 with global MAF>=5%, present in high-coverage samples sequenced at Illumina with similar allele frequency to 1000 Genomes, and reliably genotyped on several microarrays commonly in use at the time of publication, including the Illumina Human Core Exome microarray, Illumina Infinium Core Exome microarray, Illumina Omni2.5 microarray, Affymetrix Genome-Wide Human SNP Array 6.0 (now Thermo-Fisher), and the pre-GSA 23andMe v4 microarray. Next, Verogen recently announced their new “Kintelligence” product, which uses the PC-relate method (Conomos et al., 2016) to discover first-third (perhaps fourth) degree relatives in Gedmatch by genotyping 10,055 autosomal markers on a MiSeq FGx. Of these 10,055 SNPs, 9,977 (99.2%) are already present on the GSA. After adding the “reliable” kinship SNPs and the Kintelligence SNPs that were not already on the GSA, the total number of biallelic autosomal SNPs retained from the 1000 Genomes data came to 590,588.

Whole-genome sequencing typically calls variant genotypes with respect to the reference, resulting in VCF files that generally do not contain genotypes at invariant sites. This down selection strategy enabled more rapid initial benchmarking of methods in Task 3 across the hundreds of simulations described below (thousands of simulation-tool-parameter combinations). Following initial characterization, deeper analysis can be performed on specific tools and specific scenarios using a larger initial starting pool of SNPs from which to simulate.

1.2.1 Classification accuracy

Classification accuracy of all tools is well above the accuracy of guessing for all tools, using either Full or the GSA+reliable set of SNPs. Accuracy of guessing indicated by the dashed red line denotes the floor of classification accuracy which is realized if all samples are assigned to the most common class (Unrelated). However, KING shows less than 1% reduced accuracy with the Full data set at 0 error, while IBD methods (IBIS and hap-IBD) show less than 1% improvement when all SNPs are included. The figure below shows the accuracy rates of all tools split by error rates and SNP selection. At 0% error, all tools performed with > 97% accuracy. At 20% error, IBD tools perform at the same rate as accuracy of guessing (a result of classifying all individuals of “Unrelated”). KING performs similarly with the Full selection, but accuracy decreases below that of guessing when GSA+reliable SNPs are used. This finding is likely a result of KING misclassifying unrelated individuals as related due to random error generating false positive matches. For a reduced data set, this will result in a larger number of individuals being misclassified as related. These errors are classified as less than the variation between simulated data sets and are acceptable for this work.

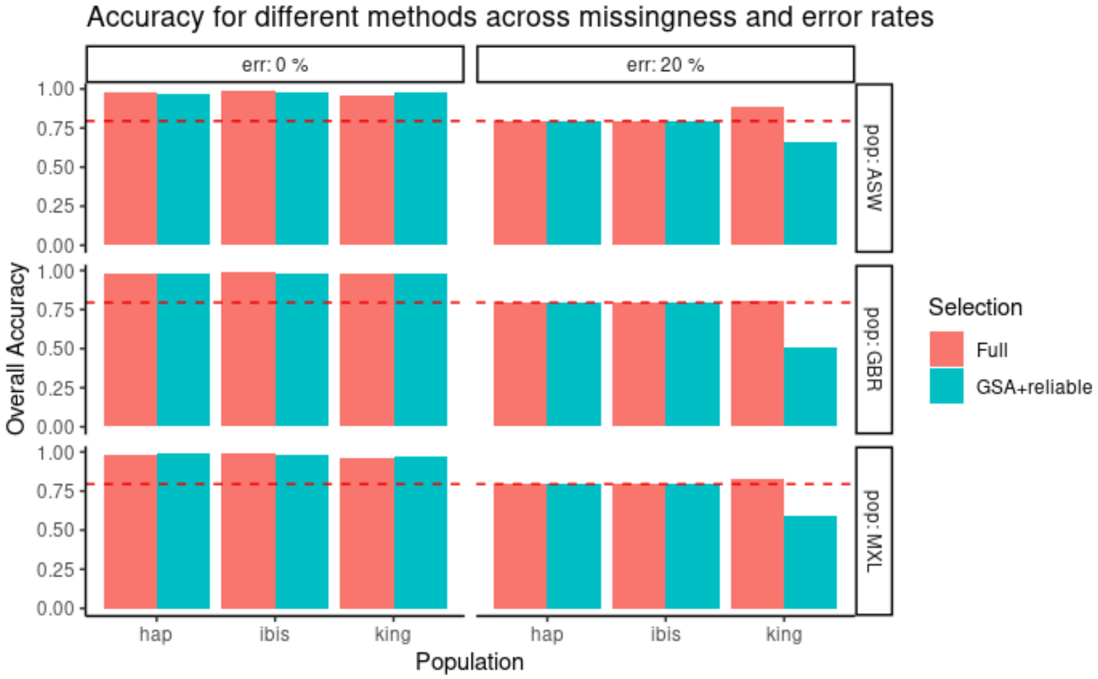

1.2.2 Kinship Coefficients

The effect of errors on KING described above (KING shows worse-than-guessing accuracy when high error is present) can be more simply demonstrated using a density plot of the kinship coefficients calculated by KING. The figure below is a density plot of all the kinship coefficients calculated by all 4 simulations, with relatedness only classified up to fourth degree relatives, with all others classified as "Unrelated". With higher error, "Unrelated" individuals have a higher average kinship coefficient than at low error, causing their kinship coefficient to rise above the threshold and be classified as fourth degree relatives. For Hap-IBD and IBIS, the calculated kinship coefficient for all individuals at 20% genotyping error is 0.

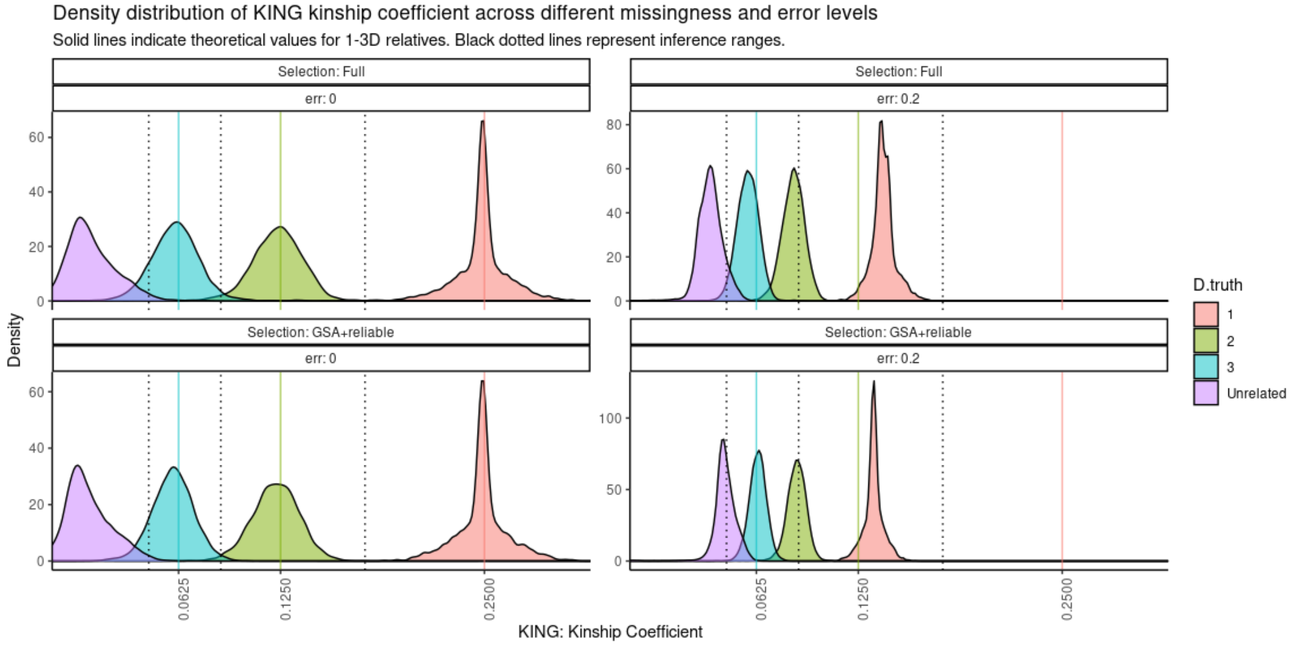

This is a provisional file, not the final typeset article

1.3 Supplementary Methods 3: data simulation with ped-sim

Initial simulations of "missing data" produced simulations with individual genotypes on all individuals in the pedigree missing at random at 1%, 5%, 10%, etc. When comparing two individuals in which each has 10% missing data, ~19% of sites will be missing in either. To capture all n, choose 2 pairwise relationships among n simulated samples while mimicking a 10% missing data rate in a single sample compared to all others, we modified the "missing data" paradigm to thinning instead of random dropout. That is, rather than simulating 10% missing at random in all n samples, we randomly thinned the entire sample by 10%. As such, each pairwise comparison of n choose 2 pairs would mimic the situation of a 10% data loss due to 10% missing in a single query sample. This effectively becomes a limit of detection kind of experiment: *how many markers are required for robust kinship estimation with different methodologies (at zero error and as genotyping error increases)*?

Ped-sim (Caballero et al., 2019) version 1.3.1 was run using a pedigree definition that resulted in the large multi-generation pedigree shown in Supplementary Figure 1. Data was simulated using the high-resolution sex-specific map from (Bhérer et al., 2017) with a crossover interference model from (Campbell et al., 2015). Unrelated individuals from each of the three populations (GBR, AFR, MXL) were used as founders to ped-sim so resulting simulated offspring genotypes would be present in the resulting VCF.

2 Supplementary Figures and Tables

2.1 Supplementary Figures

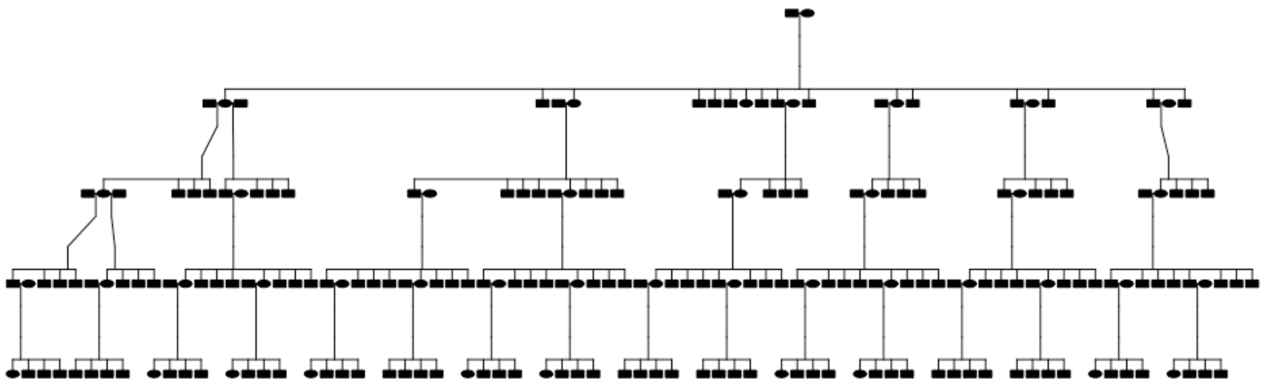

Supplementary Figure 1. Pedigree structure for each of the simulations conducted. Simulated families were defined using a pedigree definition as described in the ped-sim documentation. These relationships include parent-child (1st degree), full sibling (1st degree), avuncular (2nd degree), grandparent-grandchild (2nd degree), first cousin (3nd degree), great-great-grandparent/child (4th degree), grand-avuncular (4th degree), second cousin (5th degree), third cousin, first cousin once removed, first cousin twice removed, second cousin once removed, etc.

This is a provisional file, not the final typeset article

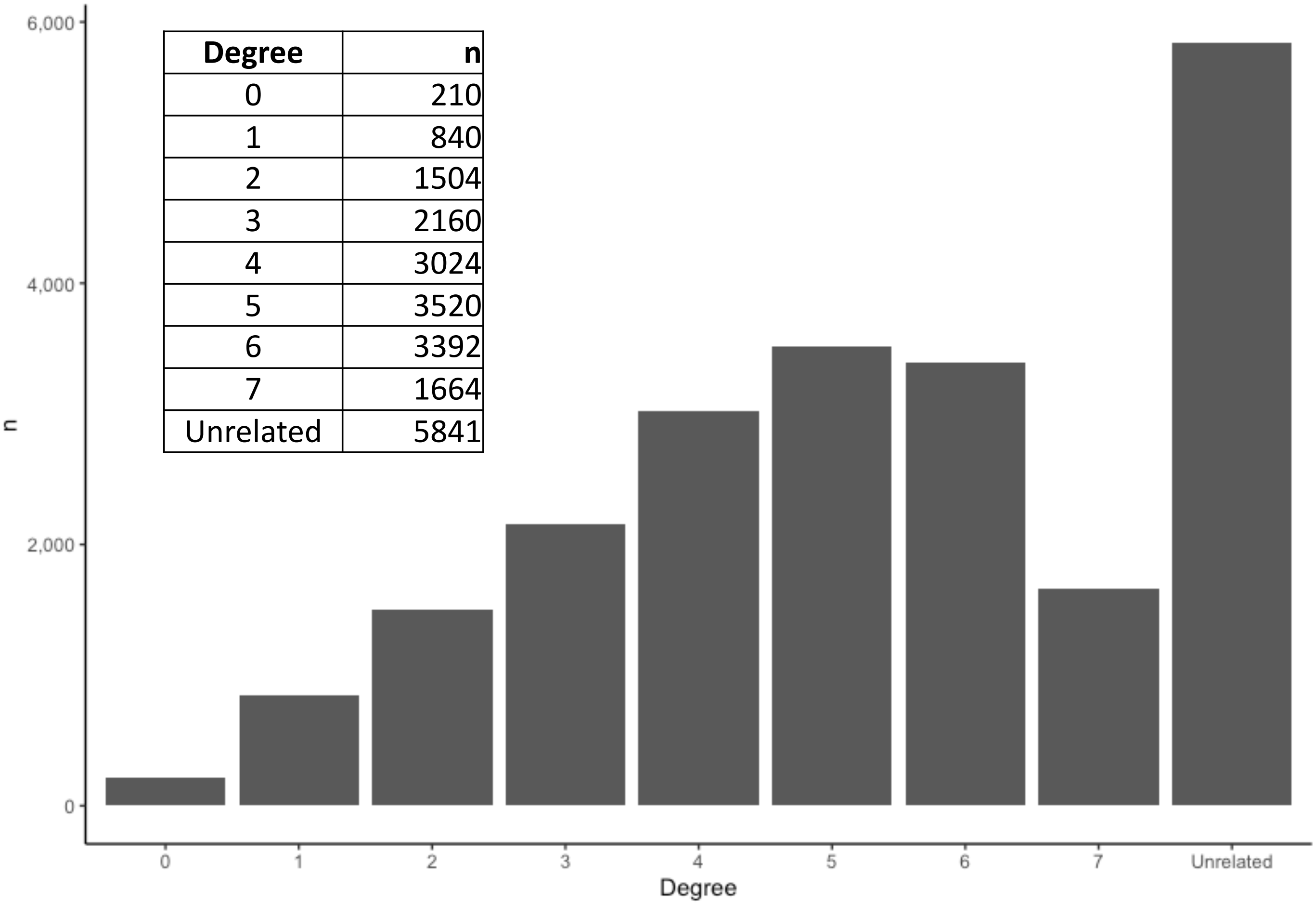

Degree	n
0	210
1	840
2	1504
3	2160
4	3024
5	3520
6	3392
7	1664
Unrelated	5841

Supplementary Figure 2: Distribution of the number of identical (0th degree), first-seventh degree relative pairs, and unrelated pairs in each of the simulations.

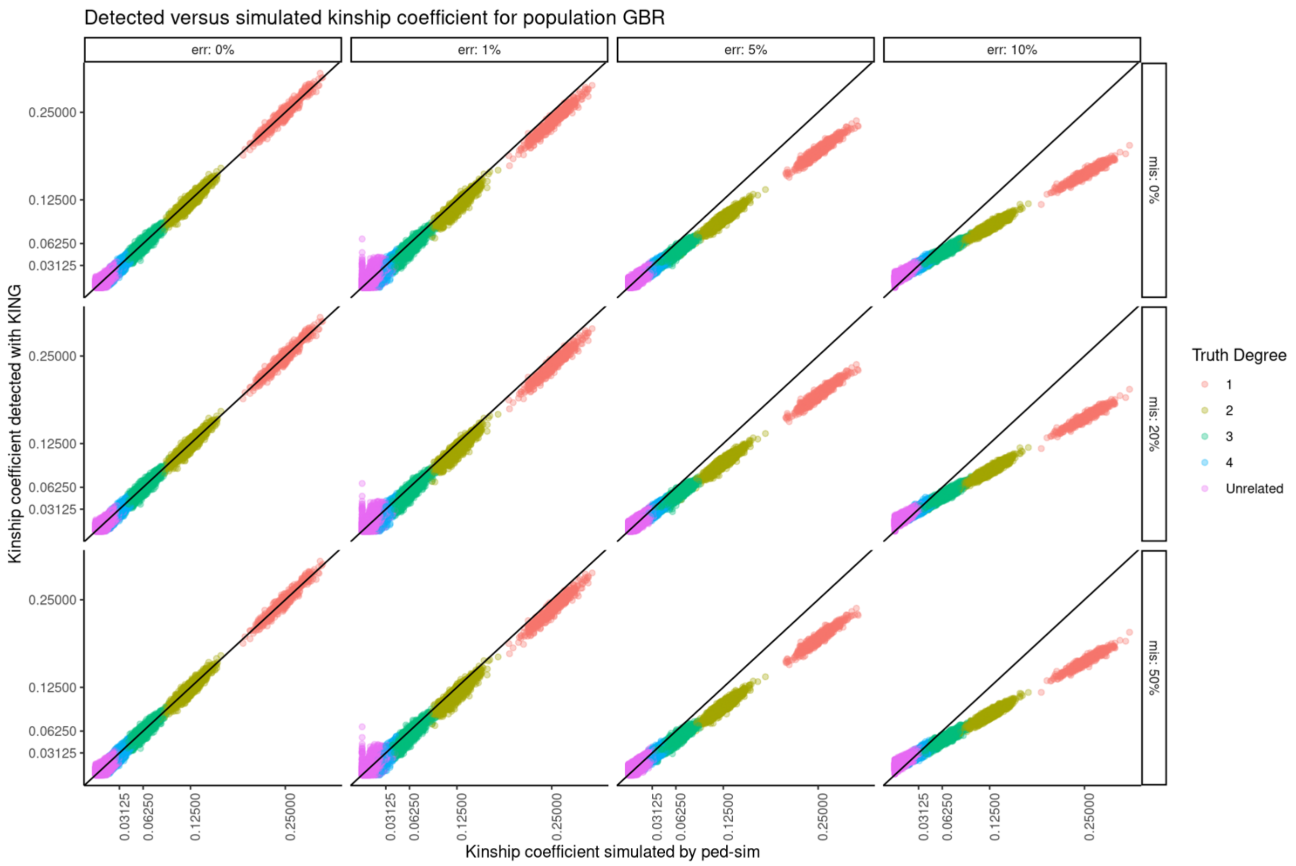

Supplementary Figure 3: Kinship coefficient inferred using KING (default parameters) against the simulated kinship coefficient for pairs of individuals simulated from GBR founders. Error increases in panels going left-to-right. Missing data increase in panels top-to-bottom. Pairs of relationships are color-coded by the true relationship degree. This graphic shows that error degrades the detected kinship coefficient, with degradation most notable in high-error simulations (5-10%).

This is a provisional file, not the final typeset article

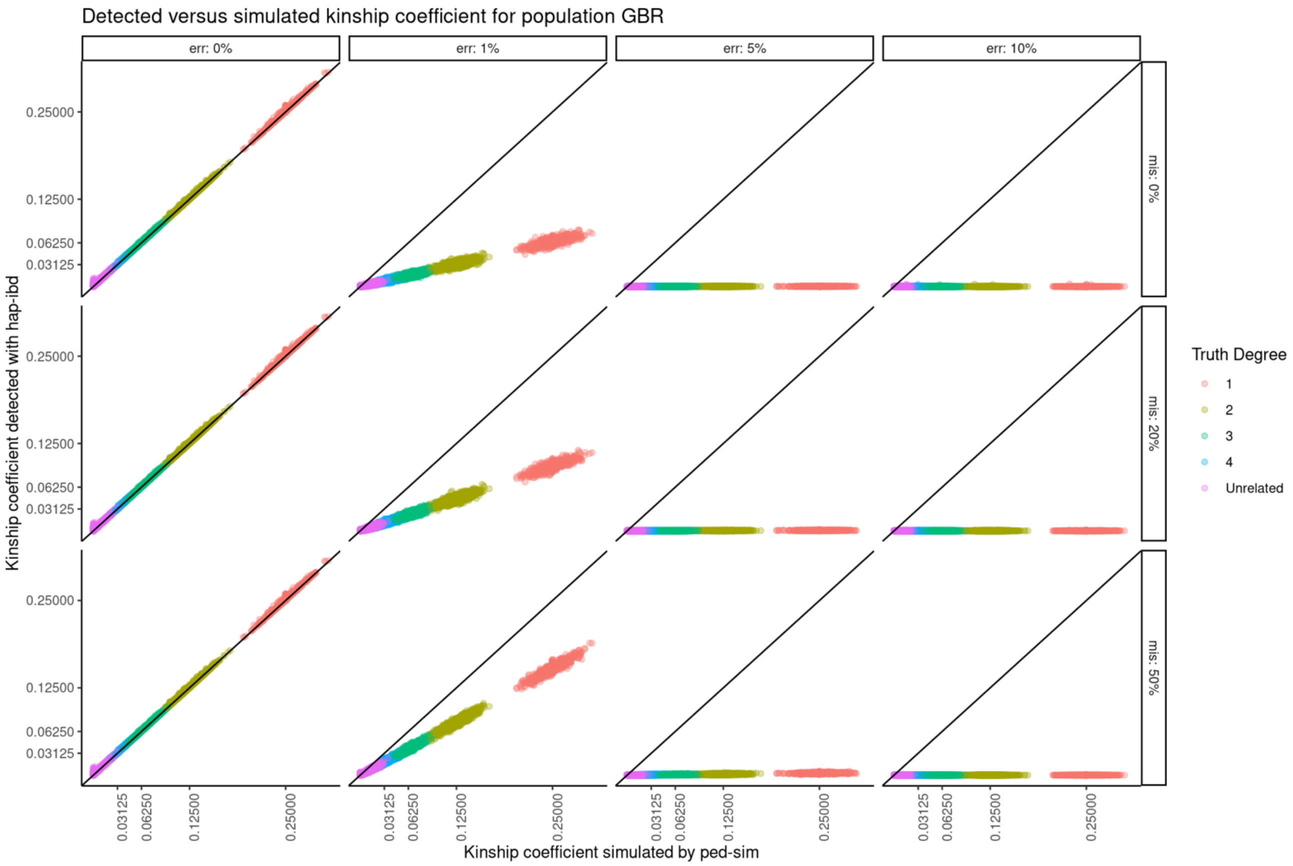

Supplementary Figure 4: Kinship coefficient inferred using hap-IBD (default parameters) against the simulated kinship coefficient for pairs of individuals simulated from GBR founders. Error increases in panels going left-to-right. Missing data increase in panels top-to-bottom. Pairs of relationships are color-coded by the true relationship degree. This graphic shows that even small amounts of genotyping error (1%) severely degrades the performance of kinship estimation and relationship inference, while larger errors (5-10%) completely eliminate hap-IBD’s ability to detect IBD segments using default parameters.

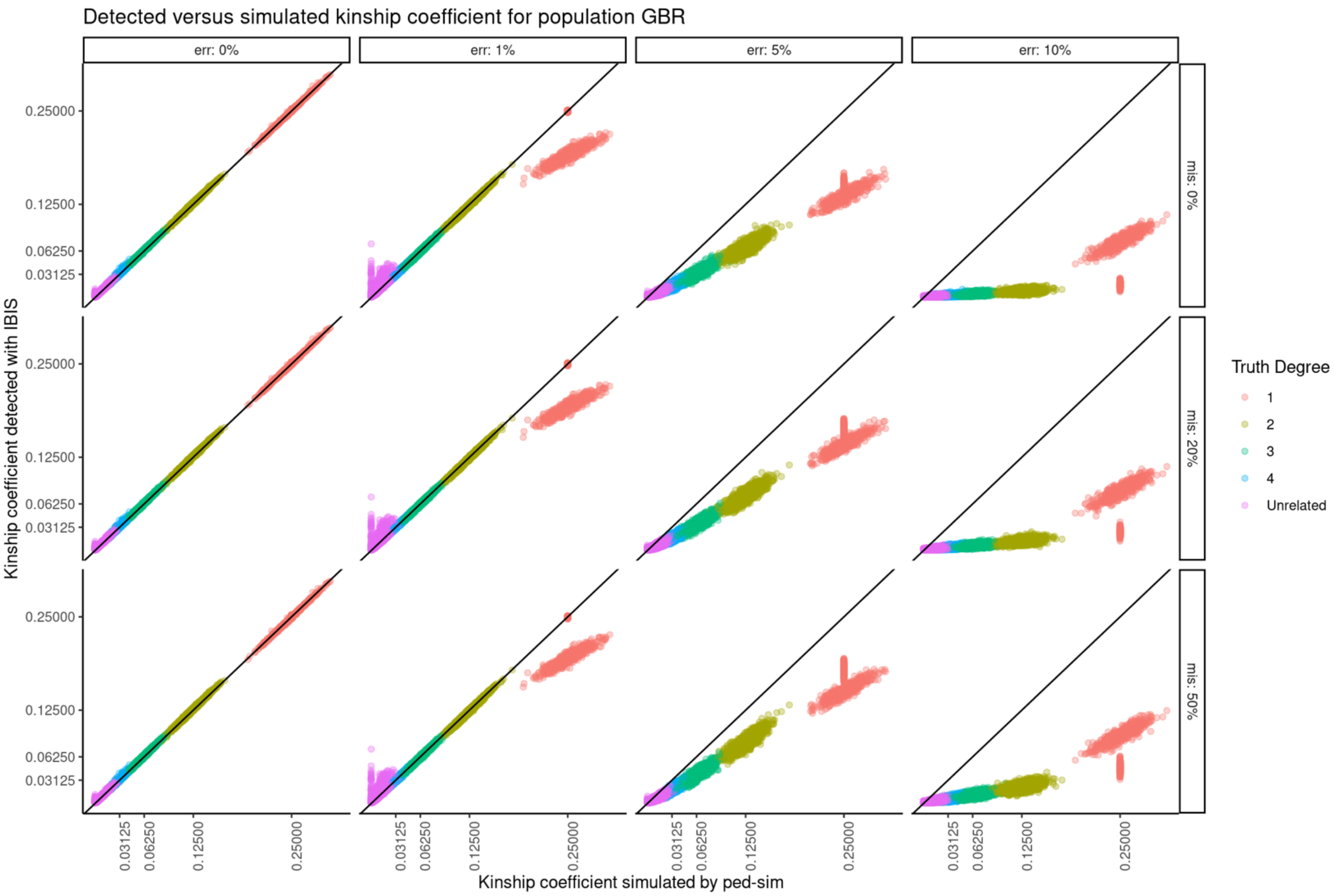

Supplementary Figure 5: Kinship coefficient inferred using IBIS (default parameters) against the simulated kinship coefficient for pairs of individuals simulated from GBR founders. Error increases in panels going left-to-right. Missing data increase in panels top-to-bottom. Pairs of relationships are color-coded by the true relationship degree. This graphic shows that relatively higher error (5-10%) severely degrades the performance of kinship estimation and relationship inference, while small errors (1%) negatively impact IBIS's ability to accurately assess full siblings, which will share on average about 25% of the genome IBD2.

This is a provisional file, not the final typeset article

2.2 Supplementary Tables

Supplementary Table 1. Simulation parameters. The 88 combinations of missingness, genotyping error, and homozygous error rate conditional upon a genotyping error were applied across founders from 1000 Genomes three populations (GBR, ASW, MXL).

Missingness rate	Genotyping error rate	Homozygous error rate
0	0	0
0	0.01	0
0	0.01	0.01
0	0.05	0
0	0.05	0.01
0	0.1	0
0	0.1	0.01
0	0.2	0
0	0.2	0.01
0	0.3	0
0	0.3	0.01
0.01	0	0
0.01	0.01	0
0.01	0.01	0.01
0.01	0.05	0
0.01	0.05	0.01
0.01	0.1	0
0.01	0.1	0.01
0.01	0.2	0
0.01	0.2	0.01
0.01	0.3	0
0.01	0.3	0.01
0.05	0	0
0.05	0.01	0
0.05	0.01	0.01
0.05	0.05	0
0.05	0.05	0.01
0.05	0.1	0
0.05	0.1	0.01
0.05	0.2	0
0.05	0.2	0.01
0.05	0.3	0
0.05	0.3	0.01
0.1	0	0
0.1	0.01	0
0.1	0.01	0.01
0.1	0.05	0
0.1	0.05	0.01
0.1	0.1	0

0.1	0.1	0.01
0.1	0.2	0
0.1	0.2	0.01
0.1	0.3	0
0.1	0.3	0.01
0.2	0	0
0.2	0.01	0
0.2	0.01	0.01
0.2	0.05	0
0.2	0.05	0.01
0.2	0.1	0
0.2	0.1	0.01
0.2	0.2	0
0.2	0.2	0.01
0.2	0.3	0
0.2	0.3	0.01
0.3	0	0
0.3	0.01	0
0.3	0.01	0.01
0.3	0.05	0
0.3	0.05	0.01
0.3	0.1	0
0.3	0.1	0.01
0.3	0.2	0
0.3	0.2	0.01
0.3	0.3	0
0.3	0.3	0.01
0.4	0	0
0.4	0.01	0
0.4	0.01	0.01
0.4	0.05	0
0.4	0.05	0.01
0.4	0.1	0
0.4	0.1	0.01
0.4	0.2	0
0.4	0.2	0.01
0.4	0.3	0
0.4	0.3	0.01
0.5	0	0
0.5	0.01	0
0.5	0.01	0.01
0.5	0.05	0
0.5	0.05	0.01
0.5	0.1	0
0.5	0.1	0.01
0.5	0.2	0
0.5	0.2	0.01

This is a provisional file, not the final typeset article

0.5	0.3	0
0.5	0.3	0.01

Supplementary Table 2. Selected Parameters for IBD tools. The KING method does not have parameters that can be tuned; however, IBIS and hap-IBD can be run with different settings to detect IBD segments within different constraints. For tools that calculate IBD segments, the most impactful measures are related to the allowed error rate to continue a segment, the minimum length of segment reported, and the maximum distance between SNPs for a segment to be continued. Selected parameters for these tools are shown below. In general, The length requirement (mL for IBIS and min-output for hap-ibd) refers to the minimal size of an identified IBD segment to keep, lowering this would allow keeping shorter segments, assuming error in the simulations break the segments. The markers required to generate a segment looks at the ability to identify a segment, again shortened to allow errors to not skip IBD segment generation. Ibis also allows for an allowable error rate, which was tested between 0.01, 0.1 and 0.2.

Tool	Parameter	Description	Selected	Total runs/tool
**IBIS**	**-ibd2**	Use IBD2 searching for IBIS	Y/N	12
	**-mL**	Minimum size of IBD kept, cM	2,7	
	**-mt**	Number of markers required for an IBD segment	<default>64, 10, 2	
	**-er2**	Allowable error rate of a segment before stopping elongation	0.01, 0.1, 0.2	
**hap-IBD**	**min-output**	Minimum output segment length	2,7	4
	**min-markers**	Minimum markers for seed of hap-IBD segments	100, 64	
	**Max-gap**	Maximum distance between two markers before segment is broken		

3 References

Arthur, R., Schulz-Trieglaff, O., Cox, A. J., and O'Connell, J. (2017). AKT: ancestry and kinship toolkit. *Bioinforma. Oxf. Engl.* 33, 142–144. doi: 10.1093/bioinformatics/btw576.

Bhérer, C., Campbell, C. L., and Auton, A. (2017). Refined genetic maps reveal sexual dimorphism in human meiotic recombination at multiple scales. *Nat. Commun.* 8, 14994. doi: 10.1038/ncomms14994.

Browning, B. L., and Browning, S. R. (2011). A Fast, Powerful Method for Detecting Identity by Descent. *Am. J. Hum. Genet.* 88, 173–182. doi: 10.1016/j.ajhg.2011.01.010.

Browning, B. L., and Browning, S. R. (2013). Improving the Accuracy and Efficiency of Identity-by-Descent Detection in Population Data. *Genetics* 194, 459–471. doi: 10.1534/genetics.113.150029.

Caballero, M., Seidman, D. N., Qiao, Y., Sannerud, J., Dyer, T. D., Lehman, D. M., et al. (2019). Crossover interference and sex-specific genetic maps shape identical by descent sharing in close relatives. *PLOS Genet.* 15, e1007979. doi: 10.1371/journal.pgen.1007979.

Campbell, C. L., Furlotte, N. A., Eriksson, N., Hinds, D., and Auton, A. (2015). Escape from crossover interference increases with maternal age. *Nat. Commun.* 6, 6260. doi: 10.1038/ncomms7260.

Chang, C. C., Chow, C. C., Tellier, L. C., Vattikuti, S., Purcell, S. M., and Lee, J. J. (2015). Second-generation PLINK: rising to the challenge of larger and richer datasets. *GigaScience* 4. doi: 10.1186/s13742-015-0047-8.

Conomos, M. P., Reiner, A. P., Weir, B. S., and Thornton, T. A. (2016). Model-free Estimation of Recent Genetic Relatedness. *Am. J. Hum. Genet.* 98, 127–148. doi: 10.1016/j.ajhg.2015.11.022.

Danecek, P., Auton, A., Abecasis, G., Albers, C. A., Banks, E., DePristo, M. A., et al. (2011). The variant call format and VCFtools. *Bioinforma. Oxf. Engl.* 27, 2156–2158. doi: 10.1093/bioinformatics/btr330.

Dimitromanolakis, A., Paterson, A. D., and Sun, L. (2019). Fast and Accurate Shared Segment Detection and Relatedness Estimation in Un-phased Genetic Data via TRUFFLE. *Am. J. Hum. Genet.* 105, 78–88. doi: 10.1016/j.ajhg.2019.05.007.

Gusev, A., Lowe, J. K., Stoffel, M., Daly, M. J., Altshuler, D., Breslow, J. L., et al. (2009). Whole population, genome-wide mapping of hidden relatedness. *Genome Res.* 19, 318–326. doi: 10.1101/gr.081398.108.

Huff, C. D., Witherspoon, D. J., Simonson, T. S., Xing, J., Watkins, W. S., Zhang, Y., et al. (2011). Maximum-likelihood estimation of recent shared ancestry (ERSA). *Genome Res.* 21, 768–774. doi: 10.1101/gr.115972.110.

International Society of Genetic Genealogy (2021). ISOGG Autosomal DNA testing comparison chart. Available at: https://isogg.org/wiki/Autosomal_DNA_testing_comparison_chart [Accessed March 22, 2021].

This is a provisional file, not the final typeset article

Korneliussen, T. S., Albrechtsen, A., and Nielsen, R. (2014). ANGSD: Analysis of Next Generation Sequencing Data. *BMC Bioinformatics* 15, 356. doi: 10.1186/s12859-014-0356-4.

Korneliussen, T. S., and Moltke, I. (2015). NgsRelate: a software tool for estimating pairwise relatedness from next-generation sequencing data. *Bioinformatics*, btv509. doi: 10.1093/bioinformatics/btv509.

Manichaikul, A., Mychaleckyj, J. C., Rich, S. S., Daly, K., Sale, M., and Chen, W.-M. (2010). Robust relationship inference in genome-wide association studies. *Bioinforma. Oxf. Engl.* 26, 2867–2873. doi: 10.1093/bioinformatics/btq559.

Naseri, A., Liu, X., Tang, K., Zhang, S., and Zhi, D. (2019). RaPID: ultra-fast, powerful, and accurate detection of segments identical by descent (IBD) in biobank-scale cohorts. *Genome Biol.* 20, 143. doi: 10.1186/s13059-019-1754-8.

Purcell, S., Neale, B., Todd-Brown, K., Thomas, L., Ferreira, M. A. R., Bender, D., et al. (2007). PLINK: A Tool Set for Whole-Genome Association and Population-Based Linkage Analyses. *Am. J. Hum. Genet.* 81, 559–575. doi: 10.1086/519795.

Ramstetter, M. D., Dyer, T. D., Lehman, D. M., Curran, J. E., Duggirala, R., Blangero, J., et al. (2017). Benchmarking Relatedness Inference Methods with Genome-Wide Data from Thousands of Relatives. *Genetics* 207, 75–82. doi: 10.1534/genetics.117.1122.

Saada, J. N., Kalantzis, G., Shyr, D., Robinson, M., Gusev, A., and Palamara, P. F. (2020). Identity-by-descent detection across 487,409 British samples reveals fine-scale population structure, evolutionary history, and trait associations. *bioRxiv*, 2020.04.20.029819. doi: 10.1101/2020.04.20.029819.

Shemirani, R., Belbin, G. M., Avery, C. L., Kenny, E. E., Gignoux, C. R., and Ambite, J. L. (2019). Rapid detection of identity-by-descent tracts for mega-scale datasets. *bioRxiv*, 749507. doi: 10.1101/749507.

Staples, J., Qiao, D., Cho, M. H., Silverman, E. K., Nickerson, D. A., and Below, J. E. (2014). PRIMUS: Rapid Reconstruction of Pedigrees from Genome-wide Estimates of Identity by Descent. *Am. J. Hum. Genet.* 95, 553–564. doi: 10.1016/j.ajhg.2014.10.005.

Staples, J., Witherspoon, D. J., Jorde, L. B., Nickerson, D. A., Below, J. E., and Huff, C. D. (2016). PADRE: Pedigree-Aware Distant-Relationship Estimation. *Am. J. Hum. Genet.* 99, 154–162. doi: 10.1016/j.ajhg.2016.05.020.

Waples, R. K., Albrechtsen, A., and Moltke, I. (2019). Allele frequency-free inference of close familial relationships from genotypes or low-depth sequencing data. *Mol. Ecol.* 28, 35–48. doi: 10.1111/mec.14954.

Zhou, Y., Browning, S. R., and Browning, B. L. (2020). A Fast and Simple Method for Detecting Identity-by-Descent Segments in Large-Scale Data. *Am. J. Hum. Genet.* 106, 426–437. doi: 10.1016/j.ajhg.2020.02.010.
